# DCP1A is an unfavorable prognostic-related enhancer RNA in hepatocellular carcinoma

**DOI:** 10.18632/aging.203593

**Published:** 2021-10-04

**Authors:** Hao Wu, Jinrui Zhang, Yi Bai, Sai Zhang, Zhixin Zhang, Wen Tong, Pinsheng Han, Bing Fu, Yamin Zhang, Zhongyang Shen

**Affiliations:** 1 First Central Clinic Institute, Tianjin Medical University, Tianjin, China; 2 Department of Hepatobiliary Surgery, Tianjin First Central Hospital, School of Medicine, Nankai University, Tianjin, China; 3 Organ Transplant Center, Tianjin First Central Hospital, School of Medicine, Nankai University, Tianjin, China

**Keywords:** hepatocellular carcinoma, enhancer RNA, DCP1A, prognosis, bioinformatics

## Abstract

Long non-coding RNAs (lncRNAs) are associated with occurrence and development of tumors. Enhancer RNA (eRNA) is a special type of lncRNAs produced from transcription of enhancer elements. The function of eRNAs in tumors have elicited significant attention recently. However, the clinical significance and role of eRNAs in hepatocellular carcinoma (HCC) has not been fully explored. The current study sought to explore the expression level and prognostic value of key eRNAs in HCC. Bioinformatics analyses were used to explore expression levels of key prognostic eRNAs in HCC and their correlation with target genes. A total of 1580 enhancer RNAs (eRNAs) and 1791 target genes were initially retrieved from TCGA-LIHC gene expression database. Further analysis through survival and correlation analysis led to identification of 12 eRNAs and 13 target genes. The findings showed that DCP1A was the most prognosis-related eRNA. This eRNA showed the highest correlation with the target gene, PRKCD. Analysis showed that poor overall survival (OS) in HCC patients was correlated with high expression level of DCP1A (eRNA) and PRKCD (target gene). The up-regulation of DCP1A was associated with advanced tumor stage, larger tumor size and higher histological grade. The results of pan-cancer analysis showed that the expression of DCP1A was differentially expressed in 13 other types of tumor tissues and their corresponding normal tissues. This eRNA was highly expressed in digestive system tumors. Functional analysis showed that high expression level of DCP1A was implicated in multiple tumor-related signaling pathways. The findings of the current study indicated DCP1A is a novel biomarker that can be used as a potential therapeutic target for HCC patients.

## INTRODUCTION

Liver cancer is the third leading cause of cancer-related deaths after lung and colorectal cancers globally [1]. Hepatocellular carcinoma (HCC) is the most common type of primary liver cancers. Between 75-85% of all liver cancer cases are of hepatocellular carcinoma subtype. HCC accounts for half of the annual liver cancer incidences and deaths in China [2]. This implies that HCC is a significant threat to global and Chinese health.

The main treatment modalities for HCC include surgical resection, radiotherapy, molecular targeting liver transplantation, interventional and immunotherapy [3]. However, due to the insidious onset of HCC, the disease is mainly diagnosed at advanced stages thus many patients do not receive early intervention and treatment resulting in poor overall prognosis. Therefore, there is an urgent need to identify new efficient biomarkers to assess prognosis of HCC patients thus improving efficacy of treatment strategies and more elaborate follow-up plans.

Enhancer element is a segment of DNA sequence in the non-coding region. It binds to specific transcription factors thus enhancing transcription of target genes at different distances or in different directions [4]. Long non-coding RNAs (lncRNAs) are non-coding RNAs with a size more than 200 nucleotides in length, without protein-coding ability owing to lack of open-reading-frame (ORF) for coding proteins [5]. LncRNAs plays important roles in several biological processes as they can regulate DNA, RNA and proteins in multiple dimensions [6]. Enhancer RNA (eRNA) is a specific type of lncRNA produced by transcription of enhancer elements. Studies report that they play an important role in mediating target gene activation and transcription [7]. Production and activation of eRNAs in tumor cells, promotes activation of pro- or oncogenes and their downstream signaling pathways. For instance, activation of ESR1 in breast cancer enhances transcription of a series of cancer progression-associated eRNAs [8]. Moreover, proliferation of prostate cancer cell can be enhanced through selective promotion of expression of downstream androgen receptor related genes by the eRNA KLK3 [9]. These findings indicate that eRNAs play important roles in tumor development, thus they can be used as biomarkers or therapeutic targets in clinical practice.

The function of eRNAs has not been fully elucidated in in HCC. This study sought to explore expression profile and prognostic value of the eRNA in HCC. We used a unique bioinformatics screening strategy to screen the most prognosis-related eRNA. The findings showed that DCP1A is the eRNA that was most associated with survival. High DCP1A levels were correlated with poor prognosis in HCC patients. These findings indicate that, DCP1A is an independent prognostic risk factor and a potential therapeutic target for HCC. This is the first study to explore the association between DCP1A and HCC. The findings will provide a reference for further prognostic analysis and development of targeted therapy for HCC patients.

## RESULTS

### Identification of prognosis-associated eRNAs and target genes in HCC

**Figure 1:**
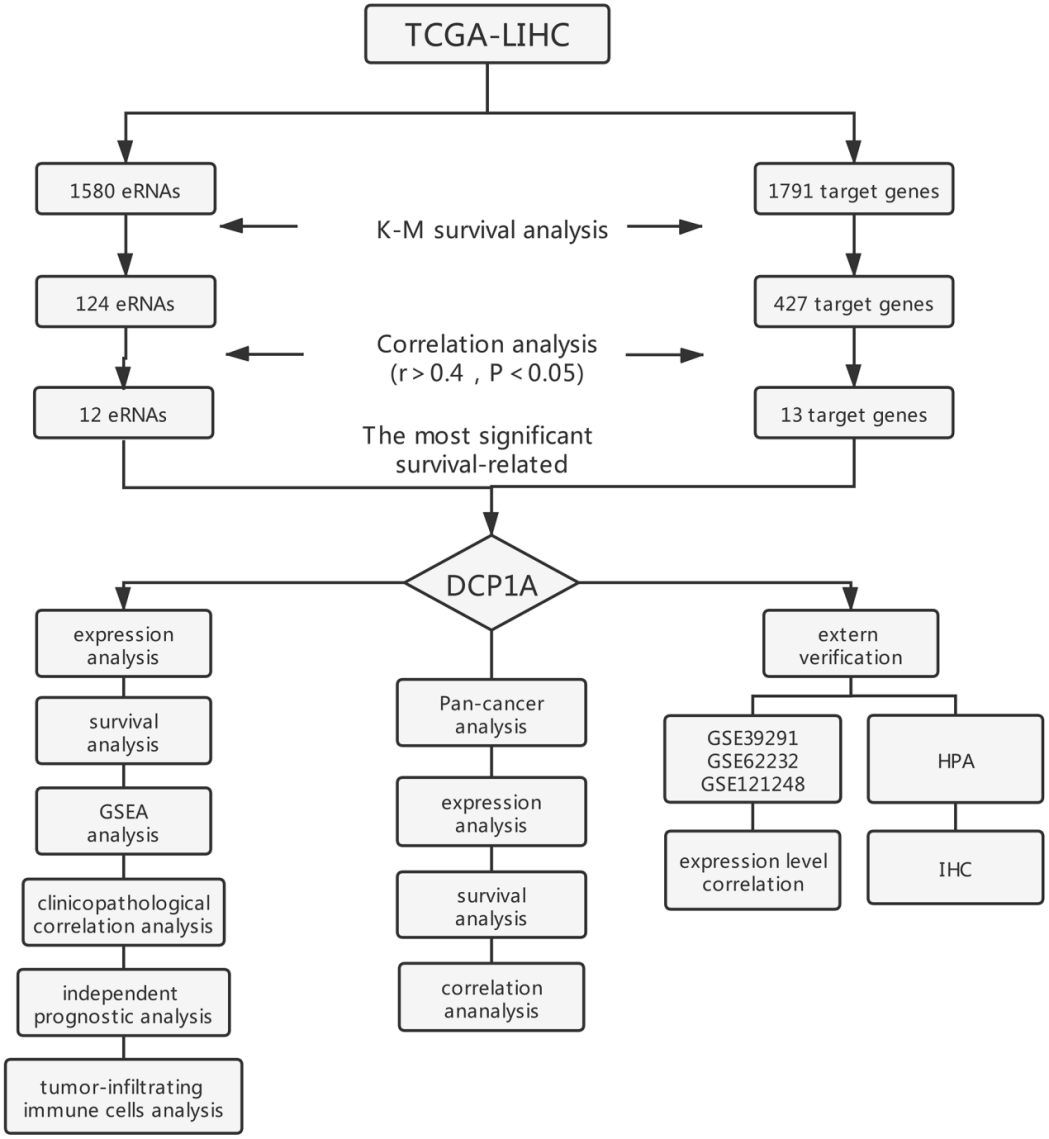
Flow chart of screening strategy for prognosis eRNA in the TCGA-LIHC cohort.

**Figure 2:**
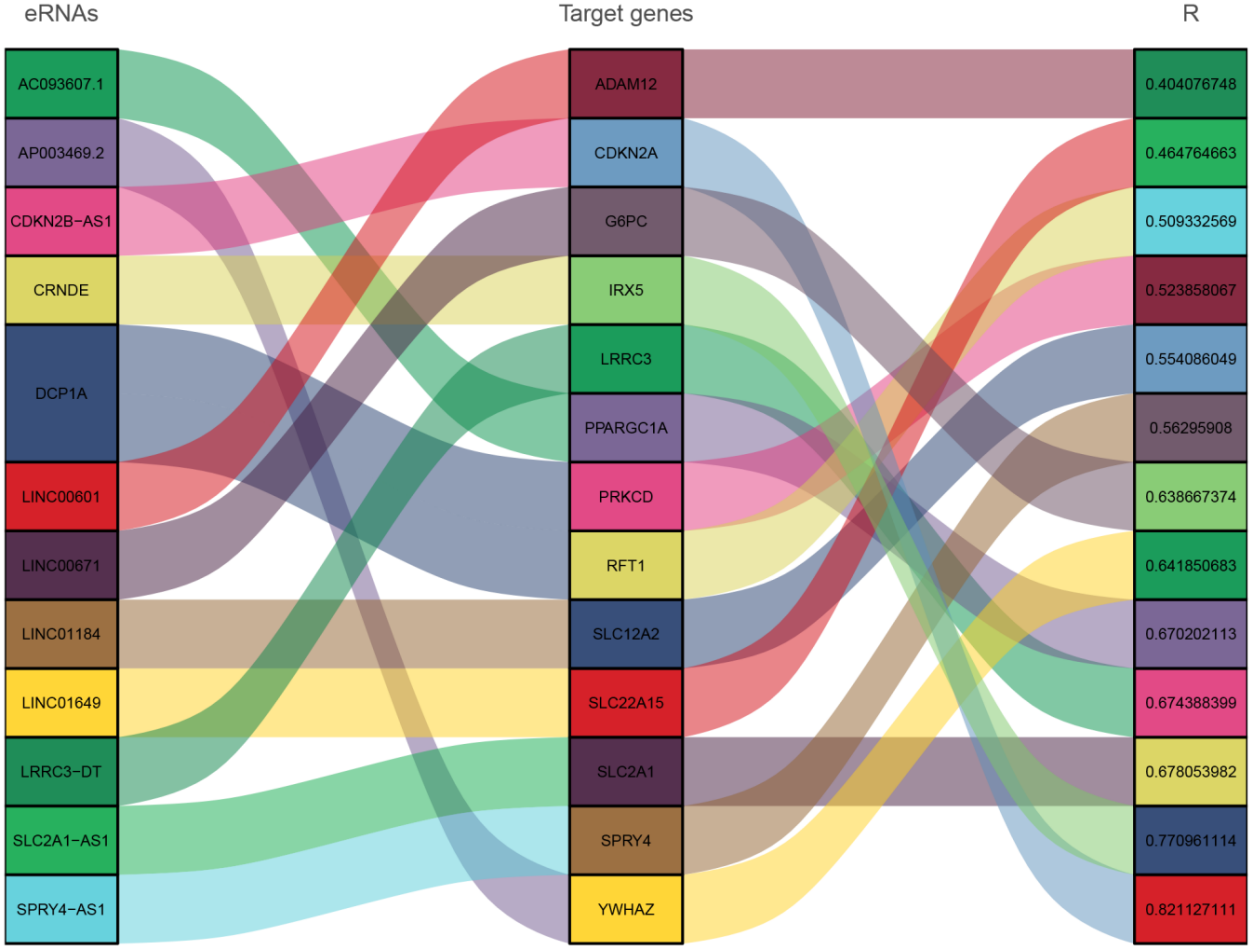
Sankey diagram showing correlation between eRNAs and target genes.

A total of 1580 eRNAs and 1791 target genes were retrieved from the gene expression data of TCGA-LIHC samples (Figure 1). Survival analysis was performed based on 368 HCC samples of the genes using Kaplan-Meier method. The findings showed that 124 eRNA and 427 target genes had prognostic significance (Kaplan-Meier and log-rank test, *P*<0.05). Further, the relationship between the eRNAs and target genes was explored through correlation analysis. The results showed a positive correlation between the expression level of the 13 pairs of genes (12 eRNAs and 13 target genes) (spearman r>0.4, *P*<0.05, Figure 2).

### DCP1A is a key eRNA in HCC

**Figure 3:**
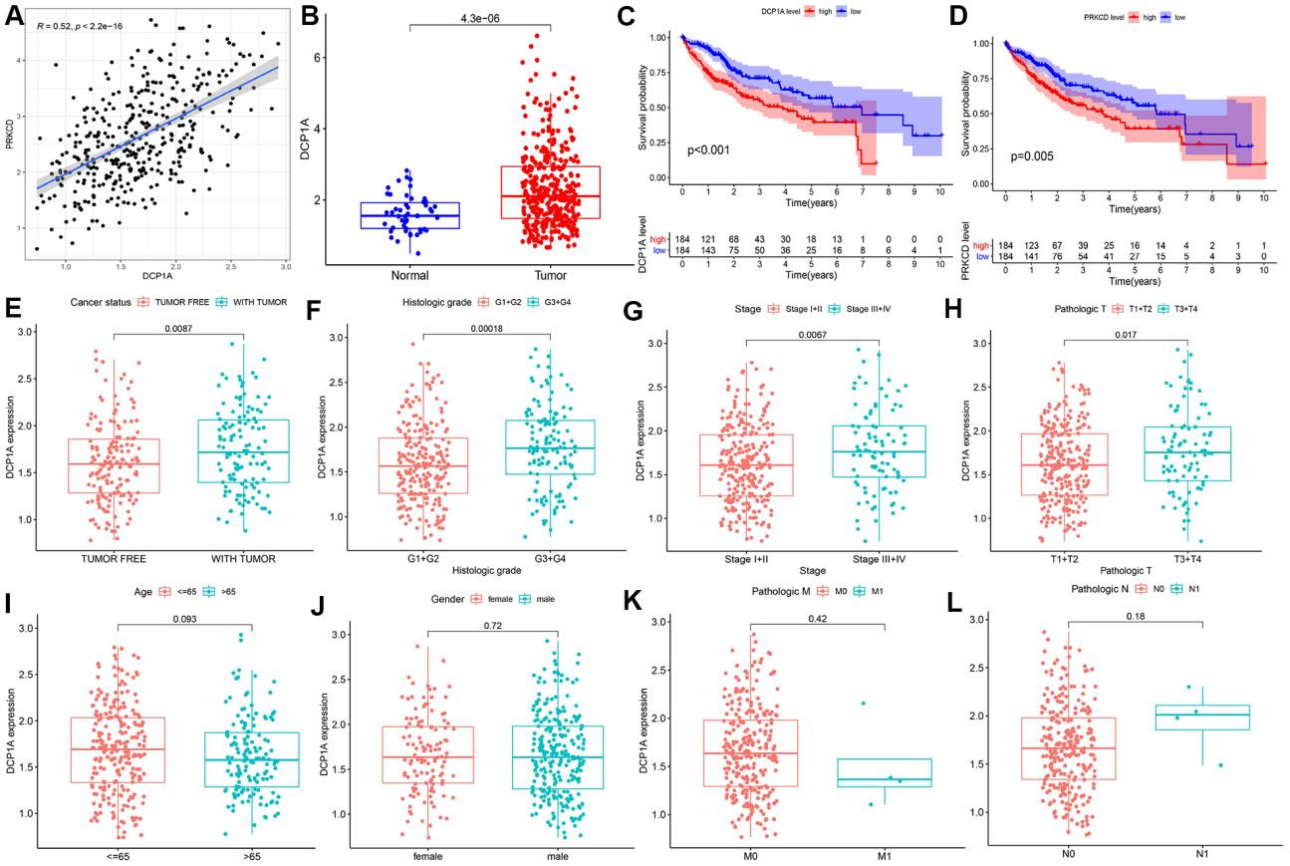
**Correlation between DCP1A and clinicopathological variables.** (**A**) Relationship between DCP1A and the predicted target gene PRKCD. (**B**) Expression level of DCP1A in HCC tissue compared with the level in normal tissue. (**C**) High expression of eRNA DCP1A was associated with poor prognosis in HCC. (**D**) High expression of PRKCD was associated with poor prognosis in HCC. (**E**–**L**) Increased expression of DCP1A was significantly correlated with cancer status, histological grade, AJCC stage, and T stage, and showed insignificant correlation with age, gender, M and N stage.

The mRNA-decapping enzyme 1a (DCP1A) was identified as the significantly associated survival eRNA with the least *P*-value among the 12 eRNAs. Expression level of DCP1A was positively correlated with expression levels of its predictive target genes, PRKCD and RFT1 (Table 1). This finding indicates that eRNAs do not only regulate specific genes, and one eRNA can regulate expression levels of multiple target genes. Therefore, the eRNA DCP1A and the target gene with a higher correlation (PRKCD) were chosen for further analysis (Figure 3A). Expression data of DCP1A were retrieved from HCC and normal samples and differential expression analysis was performed. The findings showed that DCP1A was significantly highly expressed in tumor tissues compared with the expression level in normal tissues (*P*<0.001, Figure 3B). Kaplan-Meier survival analysis showed that high expression of DCP1A was correlated with poor prognosis (*P*<0.001, Figure 3C) and the higher PRKCD expression was correlated with shorter OS (*P*=0.005, Figure 3D). The relationship between DCP1A gene expression and clinicopathological characteristics was explored. Clinical information of TCGA-LIHC cohort is presented in Table 2. The findings showed increased expression of DCP1A was significantly correlated with the cancer status (P=0.0087, Figure 3E), histological grade (G3/G4 vs G1/G2, *P*<0.001, Figure 3F), AJCC stage (stage III/IV vs stage I/II, *P*=0.0067, Figure 3G) and T stage (T3/T4 vs T1/T2, *P*=0.017, Figure 3H). However, there was no correlation between age, gender, M stage, N stage and DCP1A expression (*P*>0.05, Figure 3I–3L).

**Table 1:** Prognosis-related eRNAs and predictive target genes.

**eRNA**	**Log-rank test *P* -value**	**Target gene**	**Correlation coefficient r**	**Spearman *P*-value**
DCP1A	0.000312176	PRKCD	0.523858067	<0.001
		RFT1	0.509332569	<0.001
SLC2A1-AS1	0.000320317	SLC2A1	0.678053982	<0.001
SPRY4-AS1	0.001744478	SPRY4	0.562959080	<0.001
AP003469.2	0.004378289	YWHAZ	0.641850683	<0.001
CDKN2B-AS1	0.00745988	CDKN2A	0.821127111	<0.001
AC093607.1	0.011502567	PPARGC1A	0.670202113	<0.001
LINC01649	0.023746864	SLC22A15	0.464764663	<0.001
LRRC3-DT	0.027486076	LRRC3	0.674388399	<0.001
LINC00601	0.031580219	ADAM12	0.404076748	<0.001
CRNDE	0.03806139	IRX5	0.770961114	<0.001
LINC01184	0.039148893	SLC12A2	0.554086049	<0.001

**Table 2:** Clinical characteristics of subjects in the TCGA-LIHC cohort.

**Parameter**	**Type**	**Patients**
Age	<=65	235(62.83%)
	>65	138(36.9%)
	unknow	1(0.27%)
Gender	female	121(32.35%)
	male	253(67.65%)
Histologic grade	G1	55(14.71%)
	G2	178(47.59%)
	G3	124(33.16%)
	G4	12(3.21%)
	unknow	5(1.34%)
Pathologic M	M0	268(71.66%)
	M1	4(1.07%)
	unknow	102(27.27%)
Pathologic N	N0	254(67.91%)
	N1	4(1.07%)
	unknow	116(31.02%)
	T2	95(25.4%)
	T3	80(21.39%)
	T4	13(3.48%)
	unknow	3(0.8%)
Cancer status	TUMOR FREE	162(43.32%)
	WITH TUMOR	124(33.16%)
	unknow	88(23.53%)
AJCC stage	Stage I	173(46.26%)
	Stage II	87(23.26%)
	Stage III	85(22.73%)
	Stage IV	5(1.34%)
	unknow	24(6.42%)

### Independent prognostic ability analysis of DCP1A

Univariate and multivariate Cox regression analyses were performed to explore the independent prognostic value of DCP1A expression and clinical characteristics. Findings from univariate analysis showed that tumor stage, T stage, M stage, and DCP1A expression were predictors of OS in HCC patients (*P*<0.05). In addition, multivariate Cox regression analyses showed that the DCP1A expression was the only independent prognostic factor for OS in HCC (*P*<0.05, Table 3). A nomogram was constructed based on DCP1A for prediction of 1-, 2-, 3-year overall survival and it was shown in Supplementary Figure 1.

**Table 3:** Univariate and multivariate Cox analysis of the correlation between DCP1A expression and OS in HCC patients.

**Parameter**	**Univariate Cox analysis**					**Multivariate Cox analysis**			
	**HR**	**HR.95L**	**HR.95H**	***P*-value**		**HR**	**HR.95L**	**HR.95H**	***P*-value**
Age	1.005	0.986	1.023	0.591		1.006	0.987	1.026	0.481
Gender	0.780	0.487	1.249	0.301		1.083	0.641	1.827	0.764
Grade	1.017	0.745	1.387	0.914		1.002	0.710	1.414	0.988
stage	1.864	1.455	2.388	8.07E-07		1.176	0.445	3.104	0.742
T stage	1.804	1.434	2.270	4.73E-07		1.477	0.612	3.560	0.385
M stage	3.849	1.206	12.281	0.022		1.706	0.441	6.591	0.438
N stage	2.021	0.493	8.276	0.327		1.508	0.224	10.110	0.672
DCP1A	1.366	1.133	1.647	0.001		1.297	1.055	1.594	0.013

### DCP1A is implicated in multiple cancer-related signaling pathways

**Figure 4:**
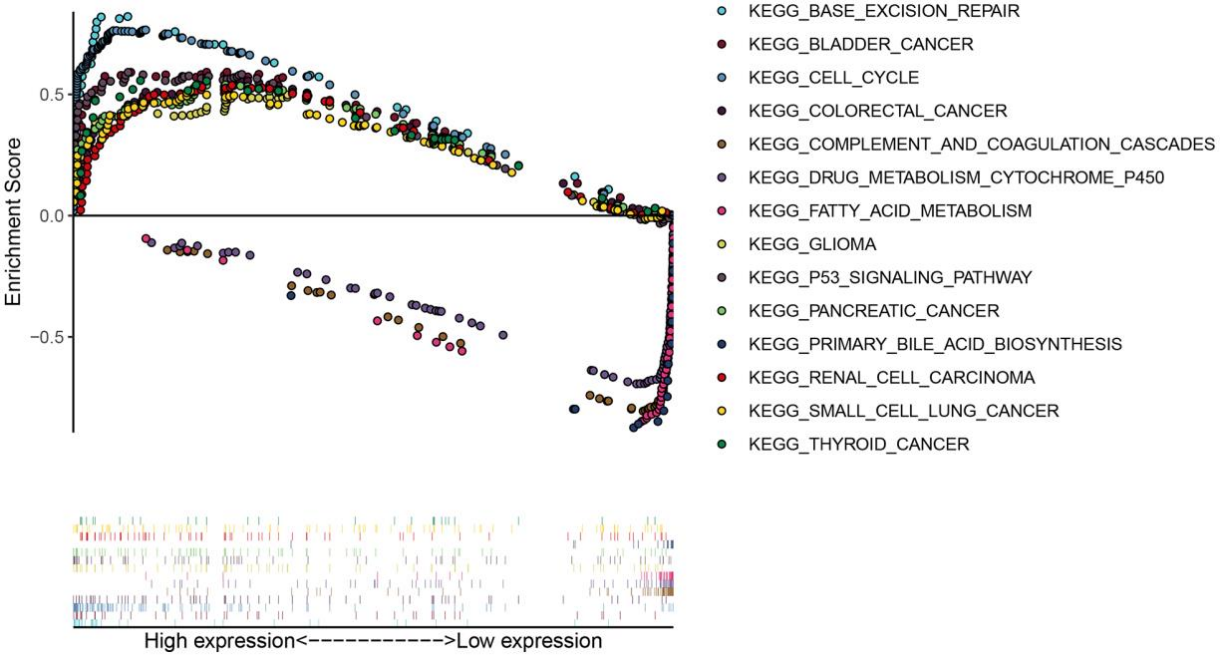
GESA analysis of DCP1A.

Gene set enrichment analysis (GSEA) analysis was performed to explore potential KEGG pathways between DCP1A high and low expression groups. The findings showed that multiple cancer-related pathways, such as “bladder cancer”, “glioma”, “pancreatic cancer”, “colorectal cancer”, “small cell lung cancer”, “thyroid cancer”, “renal cell carcinoma” and “p53 signaling pathway” were enriched in DCP1A high expression group. In addition, “base excision repair”, “cell cycle” pathways were enriched in DCP1A high expression group. DCP1A low expression group showed significant enrichment of metabolism-related pathways (Figure 4 and Supplementary Figure 2).

### Pan-cancer analysis of DCP1A

**Figure 5:**
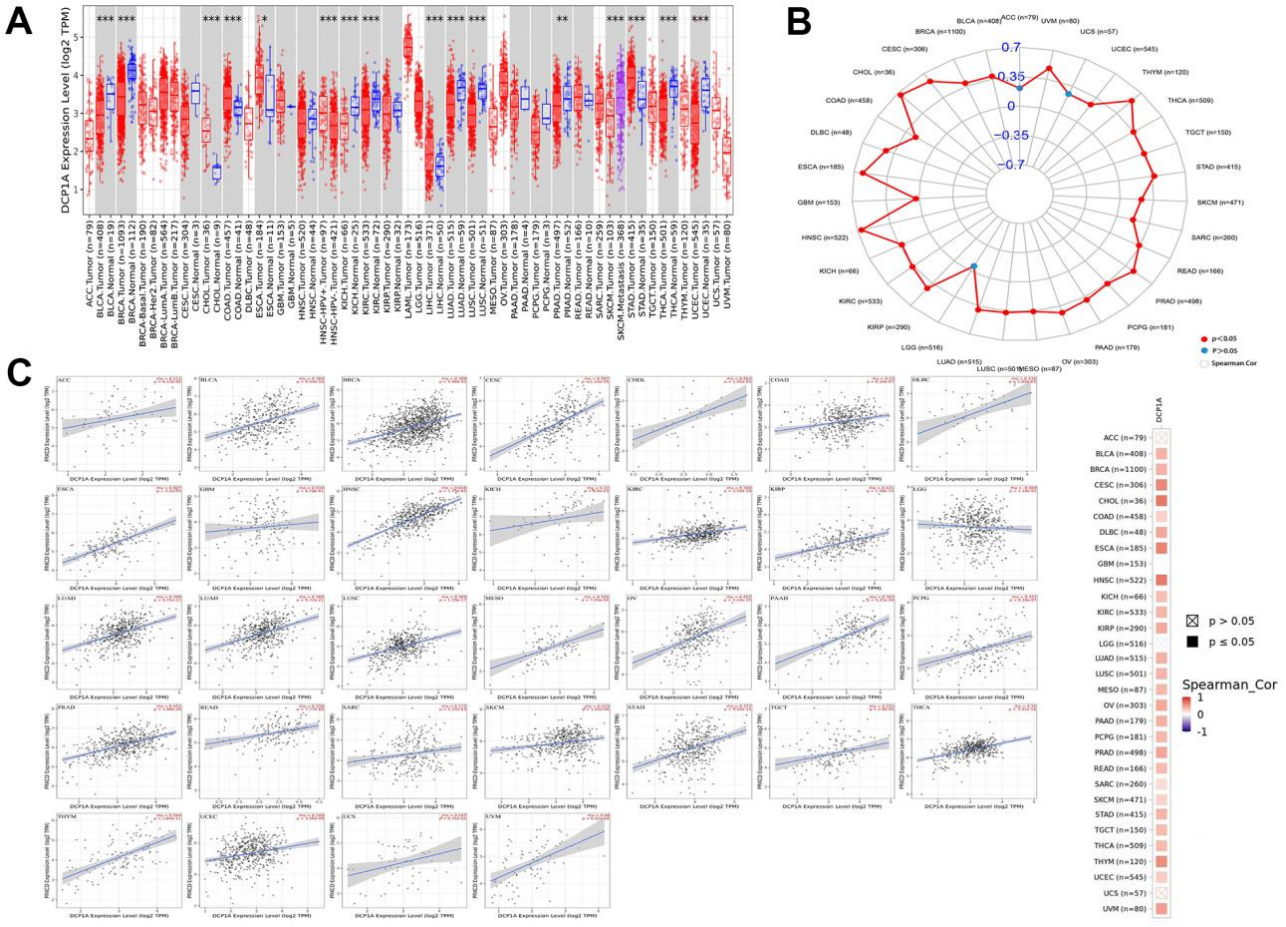
**DCP1A expression level and correlation with the target gene PRKCD in pan-cancer analysis.** (**A**) DCP1A expression in pan-cancer (*p < 0.05; **p < 0.001; ***p < 0.0001). (**B**, **C**) The correlation of DCP1A and PRKCD in pan-cancer analysis.

A pan-cancer analysis was carried out using the 32 other types cancers data from TCGA as the internal verification to further investigate the expression of DCP1A in a variety of cancers and its correlation with the target gene PRKCD. The expression of DCP1A was differentially expressed in 13 other types of tumor tissues and its corresponding normal tissues (*P*<0.05; Figure 5A). Four of the other cancers (cholangiocarcinoma CHOL), colon adenocarcinoma (COAD), esophageal carcinoma (ESCA) and stomach adenocarcinoma (STAD) showed a high expression of DCP1A as compared with normal tissues. Low DCP1A expression was evident in 9 of the other cancer tissues, including bladder urothelial carcinoma (BLCA), breast invasive carcinoma (BRCA), kidney chromophobe (KICH), kidney renal clear cell carcinoma (KIRC), lung adenocarcinoma (LUAD), prostate adenocarcinoma (PRAD), thyroid carcinoma (THCA), lung squamous cell carcinoma (LUSC) and uterine corpus endometrial carcinoma (UCEC).

The correlation between eRNA DCP1A and its predicted target gene PRKCD in different types of cancer was also verified. It was evidently noted that there was a positive correlation between DCP1A and PRKCD in all cancers, except adrenocortical carcinoma (ACC), uterine carcinosarcoma (UCS) and brain lower grade glioma (LGG) (Figure 5B, 5C).

**Figure 6:**
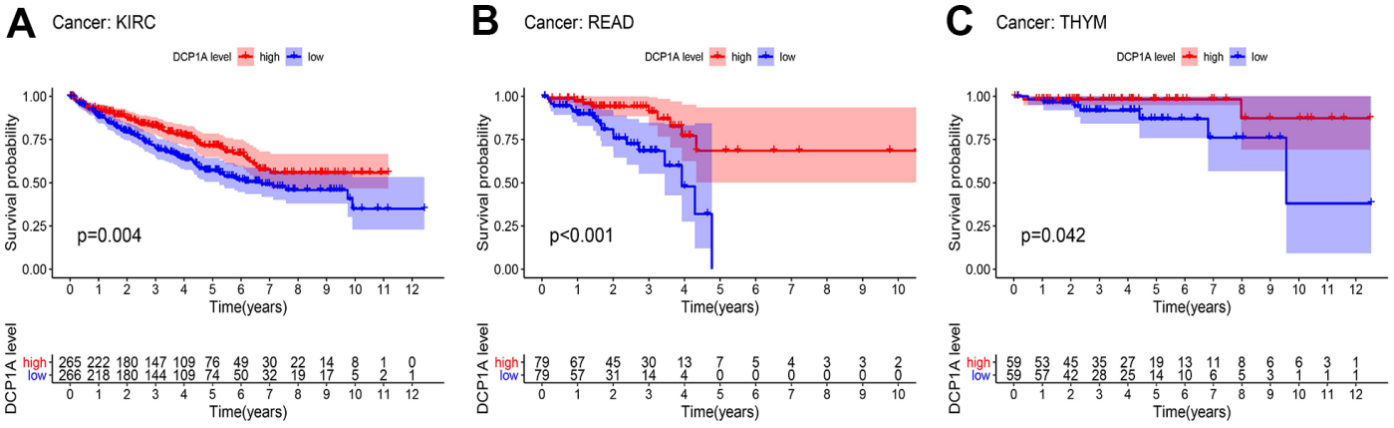
**Kaplan-Meier survival analysis of DCP1A in pan-cancer.** (**A**) DCP1A down-regulation is correlated with poor prognosis in KIRC. (**B**) DCP1A down-regulation is correlated with poor prognosis in READ. (**C**) DCP1A down-regulation is correlated with poor prognosis in THYM.

The prognostic significance of DCP1A in pan-cancer data was explored using Kaplan-Meier and log-rank tests (Supplementary Table 1). The patients were divided into high and low expression groups according to the median of DCP1A expression level. The DCP1A has prognostic significance in kidney renal clear cell carcinoma (KIRC), rectum adenocarcinoma (READ) and thymoma (THYM) (Figure 6), the high expression of DCP1A were associated with the observed favorable prognosis.

### External verification of DCP1A expression and correlation with predictive target gene PRKCD

**Figure 7:**
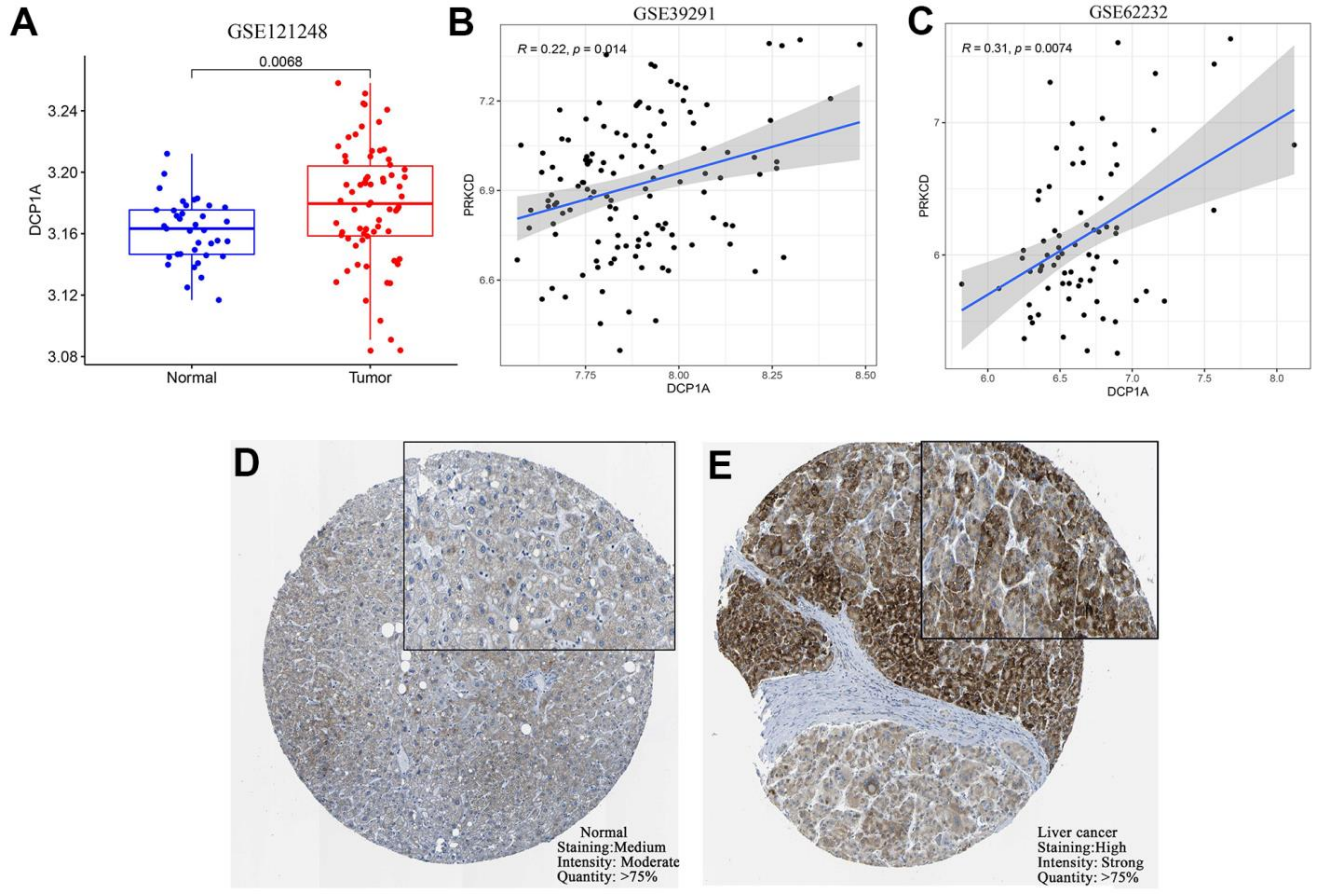
**External verification of DCP1A expression and the relationship with the target gene, PRKCD.** (**A**) Expression level of DCP1A was higher in HCC tissue compared with the level in normal tissue in the GSE121248 dataset. (**B**, **C**) DCP1A expression was positively correlated with the expression of the target gene, PRKCD in GSE39291 and GSE62232 datasets. (**D**, **E**) Expression of DCP1A was higher in HCC tissue compared with the level in normal tissue from HPA IHC dataset.

GSE121248, GSE39291, and GSE62232 datasets were retrieved from GEO database for external validation of the differential expression of DCP1A between HCC tissues and adjacent normal tissues and to further explore the correlation between DCP1A expression level and the target gene, PRKCD. Analysis showed that DCP1A was highly expressed in liver cancer tissues and its expression level was positively correlated with expression of PRKCD (*P*<0.05, Figure 7A–7C) in which was consistent with the findings using TCGA dataset. Moreover, IHC data of DCP1A in HCC tissues and normal tissues were retrieved from HPA. Analysis of IHC data showed that DCP1A protein was highly expressed in HCC tissues (Figure 7D, 7E).

### DCP1A and tumor-infiltrating immune cells

**Figure 8:**
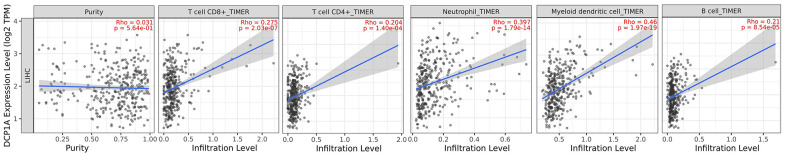
Relationship between DCP1A expression level and tumor-infiltrating immune cells.

TIMER 2.0 (http://timer.cistrome.org/) was used to explore the relationship between DCP1A expression level and abundance of tumor-infiltrated immune cells (CD4+ T cells, CD8+ T cells, B cells, neutrophils, dendritic cells). The findings showed a positive correlation between DCP1A expression level and abundance of different type of immune cells (*P*<0.05, Figure 8).

## DISCUSSION

Although the treatment of patients with HCC has been rapidly improved in the past few decades, the morbidity and mortality of HCC is still high. This is due to late diagnosis and limited accuracy of biomarkers for diagnosis and prognosis [10]. Therefore, there is an urgent need to identify new markers for prognosis and treatment of HCC to improve the survival rate of HCC patients. Several studies have demonstrated that lncRNAs play an important role in occurrence and development of HCC [11, 12]. Notably, eRNAs are a special subclass of lncRNAs, which modulate the transcription of genes, and are correlated with the prognosis of tumor, implying that eRNAs can be used as a novel therapeutic target for cancer [13, 14].

Several studies have explored the relationship between eRNAs and their prognosis value in ovarian cancer, head and neck squamous cell carcinoma, colon cancer and renal clear cell carcinoma [15–18]. However, the relationship between eRNAs and prognosis of hepatocellular carcinoma has not been fully elucidated. Therefore, the present study provides important insights into understanding the potential role of eRNA DCP1A in HCC. The study identified the eRNA that is most associated with prognosis of hepatocellular carcinoma and its target gene. A total of 1580 eRNAs and 1791 target genes were extracted from LIHC gene expression data. Kaplan-Meier survival analysis was performed to identify eRNAs and target genes correlated with survival of patients with HCC. Correlation analysis was conducted among the survival-related genes and 12 eRNA and 13 target genes were identified, and the findings also showed that some eRNAs can modulate multiple target genes.

DCP1A was the most significant survival-associated eRNA with the least *P*-value. The poor OS in HCC patients was correlated with high expression level of DCP1A and the target gene, PRKCD. Furthermore, high DCP1A expression was correlated with unfavorable clinical features, such as advanced tumor stage, tumor size (T stage), and higher histological grade. Univariate and multivariate Cox analysis were performed to verify the independent prognostic value of DCP1A. Findings from Cox analysis showed that high expression level of DCP1A was an independent prognostic risk factor for patients with HCC.

A pan-cancer analysis was performed to explore the possible role of DCP1A in other cancer types. The findings showed differential expression of DCP1A between 13 different types of cancer and the corresponding normal tissues. Expression of DCP1A was up-regulated in four tumor types (CHOL, COAD, ESCA, and STAD) whereas DCPIA expression was significantly down-regulated in 9 of tumor types. Moreover, analysis showed that all the four tumor tissues (CHOL, COAD, ESCA, and STAD) with high expression level of DCP1A were digestive system tumors.

The results of pan-cancer survival analysis showed that the expression level of DCP1A was associated with the OS of patients with KIRC, READ, and THYM. This finding indicated that patients with high expression level of DCP1A had favorable prognosis. In addition, DCP1A was positively correlated with expression of PRKCD in all cancers except ACC, USC, and UGG. These findings imply that DCP1A acts as eRNA to promote expression of the target gene, PRKCD.

The findings of the current study indicated that DCP1A plays a role as tumor suppressor gene and oncogene in different types of tumors. DCP1A plays a role as an oncogene in digestive system tumors with high expression level indicating poor prognosis thus it is a potential therapeutic target. Messenger-RNA (mRNA) plays an important role in regulation of gene expression in cells. DCP1A is a component of the decapping enzyme complex thus it is involved in regulation of mRNA removal and degradation [19, 20]. DCP1A is implicated in degradation process involved in processing bodies (P-bodies). Previous studies report that P-bodies are associated with multiple diseases, including cancer [21, 22]. Currently, only a few studies have explored the relationship between DCP1A and malignancy. A study by Ruan et al. [23] reported that high DCP1A expression level is correlated with occurrence and development of gastric cancer and prognosis of gastric cancer. Resistance of gastric cancer cells to chemotherapy can be abrogated by downregulation of DCP1A expression. Wu et al. [24] reported that DCP1A is highly expressed in colorectal carcinoma and high expression of DCP1A is associated with poor prognosis of colorectal carcinoma. Further, DCP1A is highly expressed in malignant melanoma and its high expression in malignant melanoma is correlated with poor prognosis [25].

The findings of the current study showed that DCP1A is highly expressed in HCC tissues compared with the expression level in normal tissues. Moreover, high expression level of DCP1A in HCC was correlated with poor prognosis in HCC patients. This finding implies that DCP1A is an independent unfavorable factor for HCC. To the best of our knowledge, this is the first study to explore the association between DCP1A and HCC.

Protein kinase C delta isoform (PRKCD) is a protein kinase C (PKC) family member. PRKCD activity in implicated in cell survival, proliferation and death [26]. Previous studies report that PRKCD has varied effects on different tumors [27]. For instance, PRKCD plays a role of tumor suppressor gene in breast cancer whereas it promotes tumor progression in pancreatic cancer [28, 29]. The findings of this study show that in consonance with high expression of DCP1A, the HCC patients with high expression of PRKCD also have a poor prognosis. Therefore, as the target gene of eRNA DCP1A, they could play a synergistic role in promoting the progression of liver cancer.

GSEA analysis showed that high expression level of DCP1A was correlated with significant enrichment of different of tumor-related signal pathways. The findings showed that “base excision repair”, “cell cycle”, “p53 signaling pathway” were significantly enriched in the DCP1A high expression group. This finding implies that the eRNA DCP1A may promote occurrence and progression of HCC by modulating cell cycle process and p53 signal pathway. The expression of DCP1A was also positively correlated with various tumor infiltrating immune cells. Further, results of this study also indicate that DCP1A have a significant correlation with tumor.

The current study had some limitations. The study used a small sample size thus the findings may be biased. Therefore, more studies with larger sample size should be conducted to verify the findings.

## CONCLUSIONS

The present study used bioinformatics analyses to screen a series of eRNAs and target genes correlated with prognosis in HCC patients. The eRNA DCP1A was found to be the most significant survival-associated eRNA in HCC that could serve as an independent unfavorable prognostic factor in HCC. Therefore, for the first time, the findings of the current study show that the eRNA DCP1A is a potential novel biomarker and therapeutic target for HCC patients.

## MATERIALS AND METHODS

### Data acquisition

Gene expression (HTseq-FPKM) and clinical data of liver hepatocellular carcinoma (LIHC) and 32 other cancers types was downloaded from University of California Santa Cruz (USCS) database based on TCGA (http://xena.ucsc.edu/). The RNA-seq data in GSE12148, GSE39291 and GSE62232 were downloaded from the Gene Expression Omnibus (GEO) for external validation (https://www.ncbi.nlm.nih.gov/geo/). Gene expression matrix containing eRNAs and corresponding predicted target genes in LIHC were extracted for subsequent analysis according to previous studies [30, 31].

### Identification of prognostic eRNAs and target genes in HCC

The eRNAs and target genes expression data was matched with survival data after excluding samples with incomplete clinical information. Patients were assigned to high expression group and low expression group based on the median of gene expression levels. Kaplan-Meier and log-rank test were used to identify prognostic eRNAs and target genes of HCC. *P*<0.05 was considered statistically significant. Analyses were conducted using “survival” and “survminer” packages in R software (v 4.0.2).

### Correlation analysis of eRNAs and target genes

Spearman correlation analysis was performed using R software (v 4.0.2) to explore the relationship between prognostic eRNAs and target genes. eRNAs correlated with target genes were screened using the following criteria: spearman r>0.4 and *P*<0.05. Correlated gene pairs were presented in a Sankey diagram.

### Analysis of independent prognostic and clinical characteristics of key eRNA

The most significant survival-related eRNA selected from the correlated eRNAs and target genes was used for further analysis. Univariable and multivariable analyses were performed using “survival” R package based on the selected key eRNA and the clinical data to identify independent prognostic indicators. In addition, the relationship between the key eRNA and clinical features including age, gender, stage, grade and TNM staging was evaluated.

### Gene set enrichment analysis (GSEA)

Gene set enrichment analysis (GSEA) was performed using GSEA software to explore the biological pathways associated with the identified key eRNA. Subjects were assigned into high and low expression phenotypes based on the median expression values of the key eRNA. Enrichment analysis was carried out using the default parameter settings. The random number was set at 1000 times. Gene sets with FDR<0.05 were identified as significantly enriched gene set in GSEA.

### Pan-cancer analysis and external verification

The prognostic value of key eRNA expression levels was explored in 32 other cancers. mRNA expression level of the key eRNA in a dataset of different types of cancer retrieved from TCGA database containing cancerous and normal human tissues was explored using TIMER 2.0 (http://timer.cistrome.org/). In addition, GSE121248, GSE39291 and GSE62232 datasets were retrieved from GEO database to verify the expression levels of key eRNAs in tumor and normal tissues, as well as their correlation with the target gene. Human Protein Atlas (HPA) database (https://www.proteinatlas.org/) is an online tool comprising expression profiles of a variety of human proteins. The protein expression level of the hub eRNA was compared between normal and HCC tissues using immunohistochemistry (IHC) data retrieved from the HPA database to further explore the relationship between expression levels of key eRNA and HCC.

### Estimation of correlations between the key eRNA expression level and tumor-infiltrating immune cells

Relationships between expression level of the key eRNA and tumor-infiltrating immune cells in HCC including CD8+ T cells, CD4+ T cells, neutrophils, B cells, dendritic cells and tumor purity were explored using TIMER 2.0 tool [32]. TIMER 2.0 (http://timer.cistrome.org/) is a web resource used for systematic evaluations of different immune cells in diverse cancer types. This analysis provides information on the role immune microenvironment on tumor progression.

### Statistical analysis

All statistical analyses in the current study were performed using R software (v 4.0.2). Wilcoxon rank-sum test was used to determine differences between two groups whereas Spearman’s correlation coefficient was used to explore the relationship between groups. Survival analysis was performed using Kaplan-Meier method. *P*<0.05 was considered statistically significant.

## Supplementary Material

Supplementary Figures

Supplementary Table 1
