# Supplementary material for: DCP1A is an unfavorable prognostic-related enhancer RNA in hepatocellular carcinoma
Source: Aging (Albany NY). 2021 Oct 4;13(19):23020–35. doi: 10.18632/aging.203593 (PMC8544297; doi:10.18632/aging.203593)
Supplement: Supplementary Figures [file aging-13-203593-s001.pdf]

SUPPLEMENTARY FIGURES

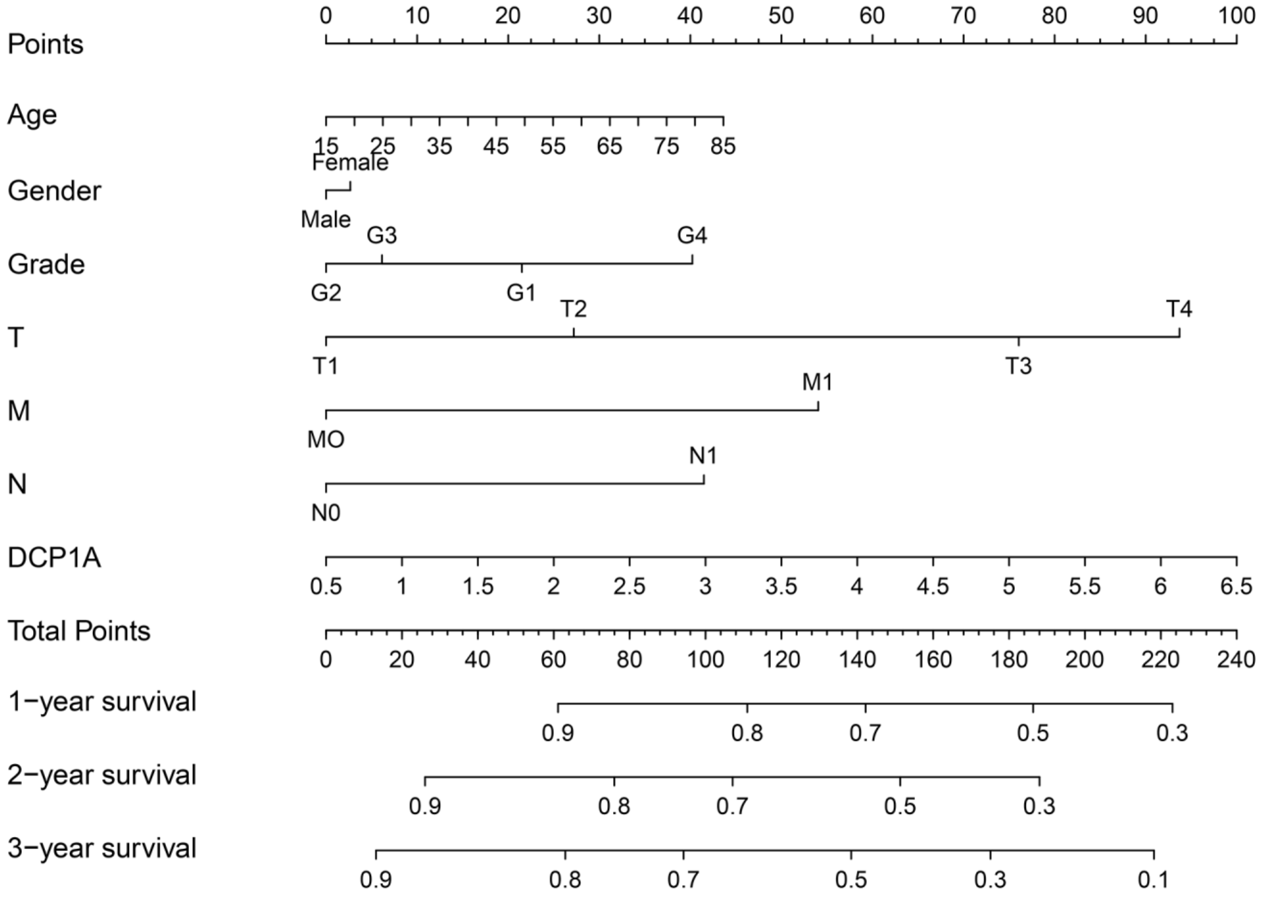

Supplementary Figure 1. Nomogram showing 1-, 2-, 3-year overall survival.

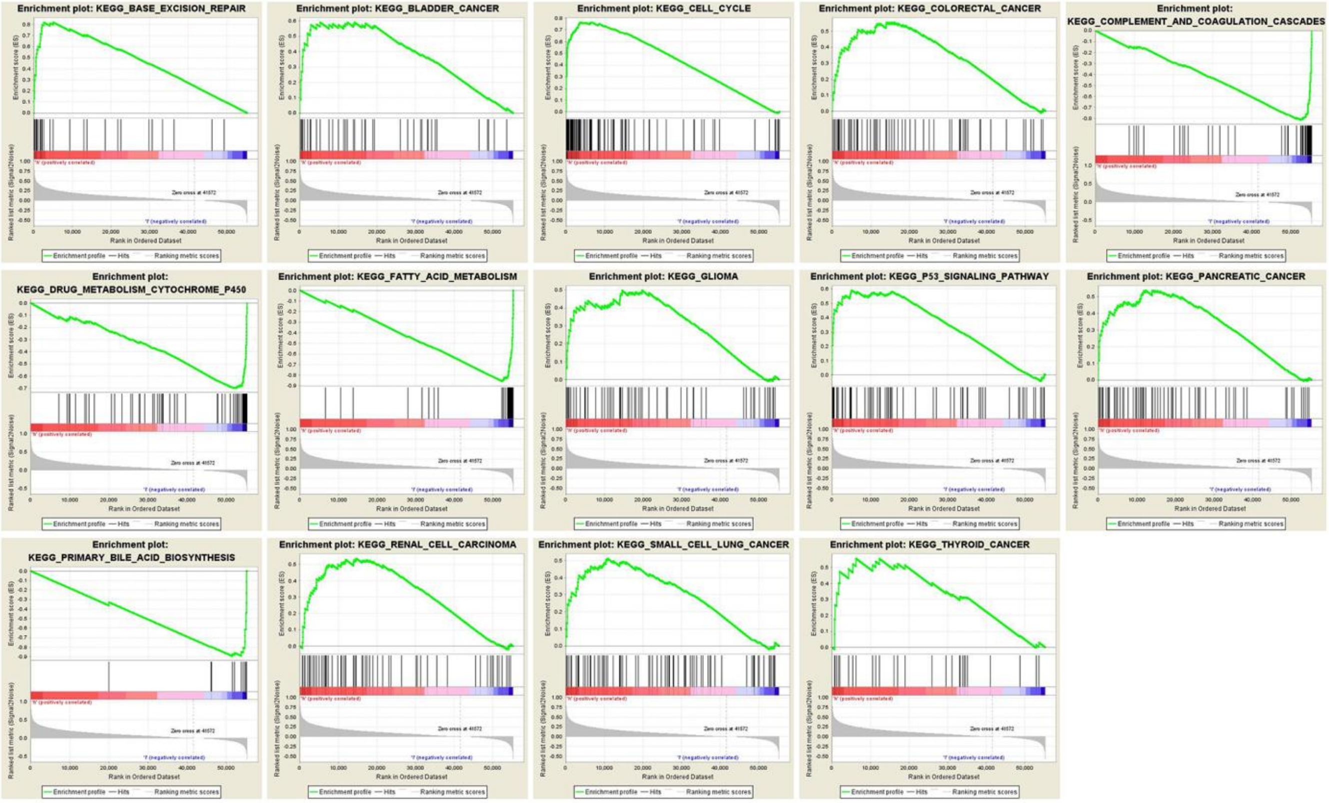

Supplementary Figure 2. GESA analysis of DCP1A. Associated with Figure 4.
