# Supplementary material for: DCP1A is an unfavorable prognostic-related enhancer RNA in hepatocellular carcinoma
Source: Aging (Albany NY). 2021 Oct 4;13(19):23020–35. doi: 10.18632/aging.203593 (PMC8544297; doi:10.18632/aging.203593)
Supplement: Supplementary Table 1 [file aging-13-203593-s002.pdf]

SUPPLEMENTARY TABLE

Supplementary Table 1. The survival results of all cancer types.

Cancer type	K-M (*P*-value)	Cancer type	K-M (*P*-value)
ACC	0.785805244	LUSC	0.152675977
BLCA	0.675132782	MESO	0.537036795
BRCA	0.386964762	OV	0.109786931
CESC	0.487109003	PAAD	0.961118896
CHOL	0.238294733	PCPG	0.418563654
COAD	0.718439838	PRAD	0.129987281
DLBC	0.711809013	READ	0.000730926
ESCA	0.709767864	SARC	0.209687067
GBM	0.321248595	SKCM	0.150320605
HNSC	0.287137528	STAD	0.312421561
KICH	0.403917932	TGCT	0.074313956
KIRC	0.003794167	THCA	0.648993352
KIRP	0.80475401	THYM	0.041583265
LAML	0.654886753	UCEC	0.26463585
LGG	0.09039115	UCS	0.803792233
LIHC	0.000312176	UVM	0.134520336
LUAD	0.065422378		
